# A Rare Presentation of a Postauricular Dermoid Cyst: A Case Report and Literature Review

**DOI:** 10.7759/cureus.107113

**Published:** 2026-04-15

**Authors:** Ethar A Alahmadi, Renad A Alhusayni, Majid A Albeladi, Syed N Naseer

**Affiliations:** 1 Otolaryngology - Head and Neck Surgery, Ohud Hospital, Medina, SAU; 2 Pathology, King Fahd Hospital, Medina, SAU

**Keywords:** benign cystic lesion, congenital dermoid cyst, head and neck mass, postauricular region, surgical excision

## Abstract

Dermoid cysts are rare congenital lesions composed of ectodermal and mesodermal elements, typically developing along embryonic fusion lines. They account for about 7% of cysts in the head and neck region, although they are uncommon in the postauricular area. This is a case of a 19-year-old male presenting with right postauricular swelling for one year. Computed tomography demonstrated a well-defined subcutaneous cyst without intracranial extension. A complete surgical excision was performed, and histopathological analysis confirmed a dermoid cyst.

Postauricular dermoid cysts present a diagnostic challenge due to their rarity and similarity to other soft-tissue swellings such as epidermoid cysts or lipomas. Computed tomography is a valuable radiological study used to assess lesion extent and exclude intracranial involvement. However, the definitive diagnosis is established through histopathological examination following surgical excision. Complete surgical excision of the cyst and its capsule prevents recurrence and ensures an excellent outcome. Postauricular dermoid cysts are exceptionally rare lesions. Complete surgical excision is both diagnostic and therapeutic and is associated with an excellent prognosis.

## Introduction

A cyst is an encapsulated cavity with clear margins that may contain a variety of materials, such as liquid, semi-liquid, or gas [1]. Dermoid cysts are a type of inclusion cyst that contains tissues from both the ectoderm and mesoderm germ layers, arising from the entrapment of ectodermal elements along the lines of embryonic closure [2].

Dermoid cysts are rare congenital cystic lesions that typically present as single, mobile, soft-tissue swellings along the midline of the body, without additional symptoms [3]. Although they can develop anywhere in the body, their occurrence in the head and neck region is uncommon, accounting for only about 7% of all cases [3]. Occurrence in the postauricular region is highly uncommon, with only a limited number of cases reported in published studies [1].

Dermoid cysts are distinguished histopathologically by identifying a squamous epithelial lining alongside skin adnexal structures, specifically hair follicles and sebaceous glands [1]. Complete surgical excision is the treatment of choice for a dermoid cyst, with an excellent prognosis and minimal risk of recurrence or complications, particularly in the absence of residual lesion or intracranial extension [2]. We report a case of a young male with a right-sided postauricular swelling that was surgically excised and evaluated for its clinical implications.

## Case presentation

A 19-year-old male with no significant past medical history presented to the otorhinolaryngology clinic with a right postauricular swelling of approximately one year. The swelling was not associated with pain, tenderness, hearing impairment, discoloration, or discharge from either the ear or the lesion. The patient denied any history of recurrent ear infections, postauricular trauma, prior postauricular surgery, or similar swellings elsewhere in the body. There were no constitutional symptoms such as fever, weight loss, or night sweats. Both personal and family history were insignificant.

**Figure 1:**
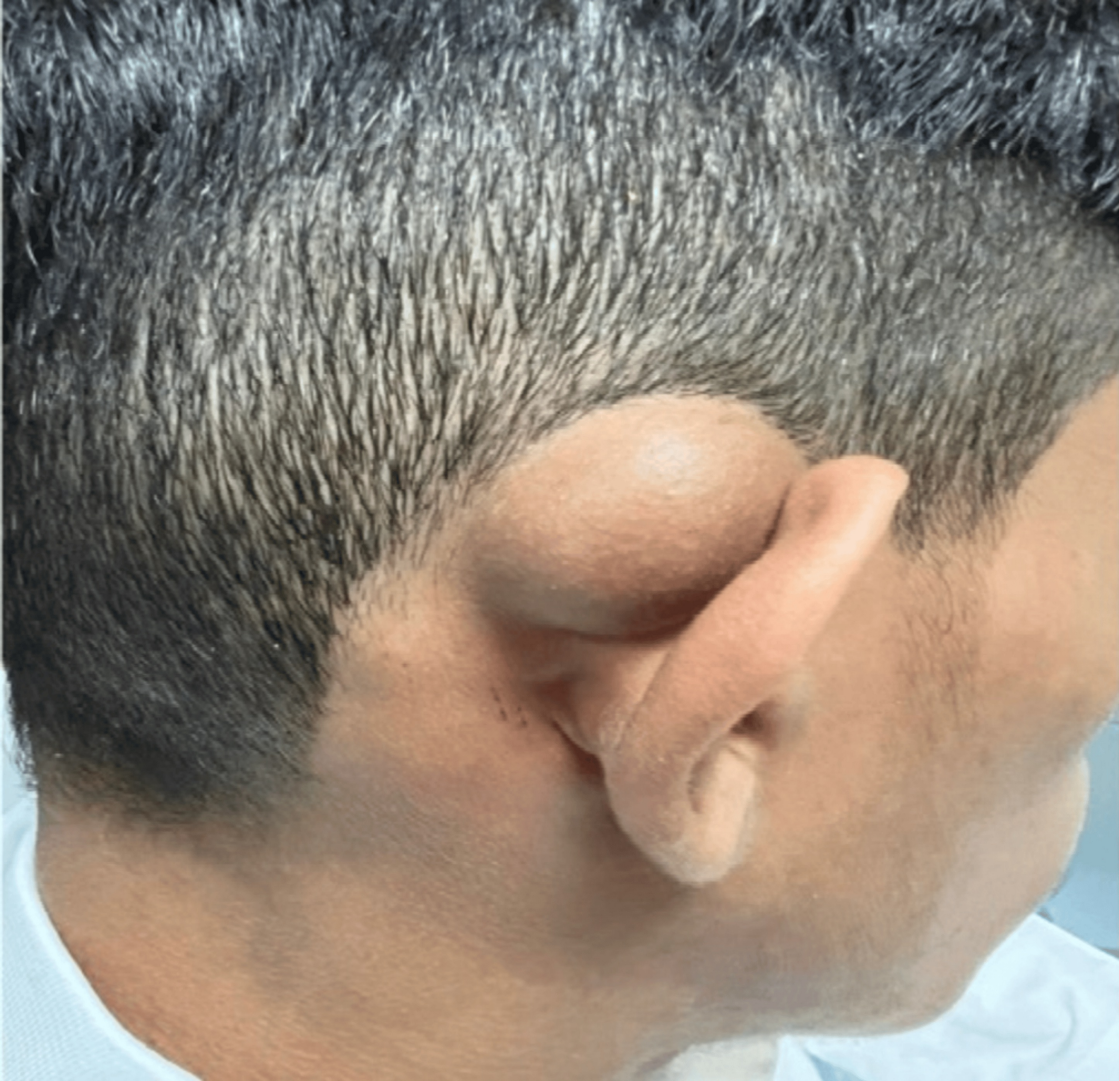
Clinical image demonstrating a well-defined, cystic mass in the right postauricular region with intact overlying skin.

On physical examination, a solitary, well-circumscribed cystic swelling was observed in the right postauricular region. The lesion extended from the upper attachment of the pinna superiorly to approximately 2 cm above the mastoid tip, causing obliteration of the retroauricular sulcus. It measured approximately 4×3×2 cm, was soft in consistency, mobile, spherical in shape, non-fluctuant, non-pulsatile, without bruit, and non-tender on palpation. The overlying skin appeared normal, with no erythema or ulceration, and there was no clinical evidence of regional lymphadenopathy (Figure 1).

**Figure 2:**
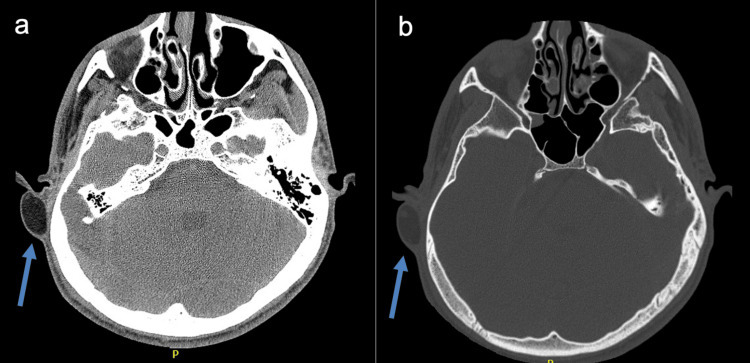
Axial computed tomography (CT) images of the temporal bone. (a) Soft-tissue window showing a well-circumscribed, low-attenuation cystic lesion in the right postauricular region, consistent with a dermoid cyst. (b) Bone window showing smooth cortical remodeling without evidence of bony erosion or intracranial extension. Blue arrows demarcate the location of the lesion.

**Figure 3:**
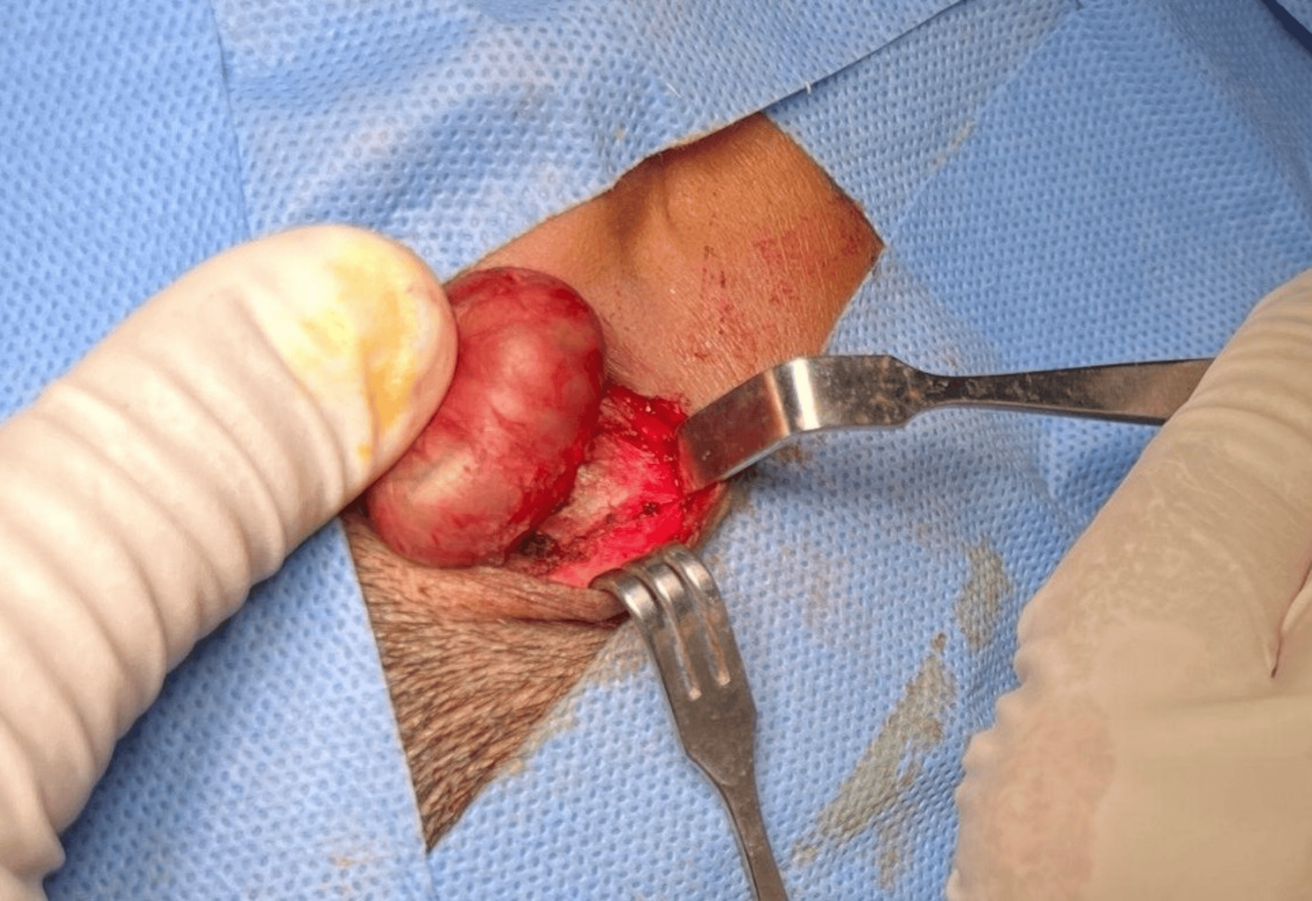
Intraoperative view showing a well-encapsulated cystic lesion in the right postauricular subcutaneous plane.

**Figure 4:**
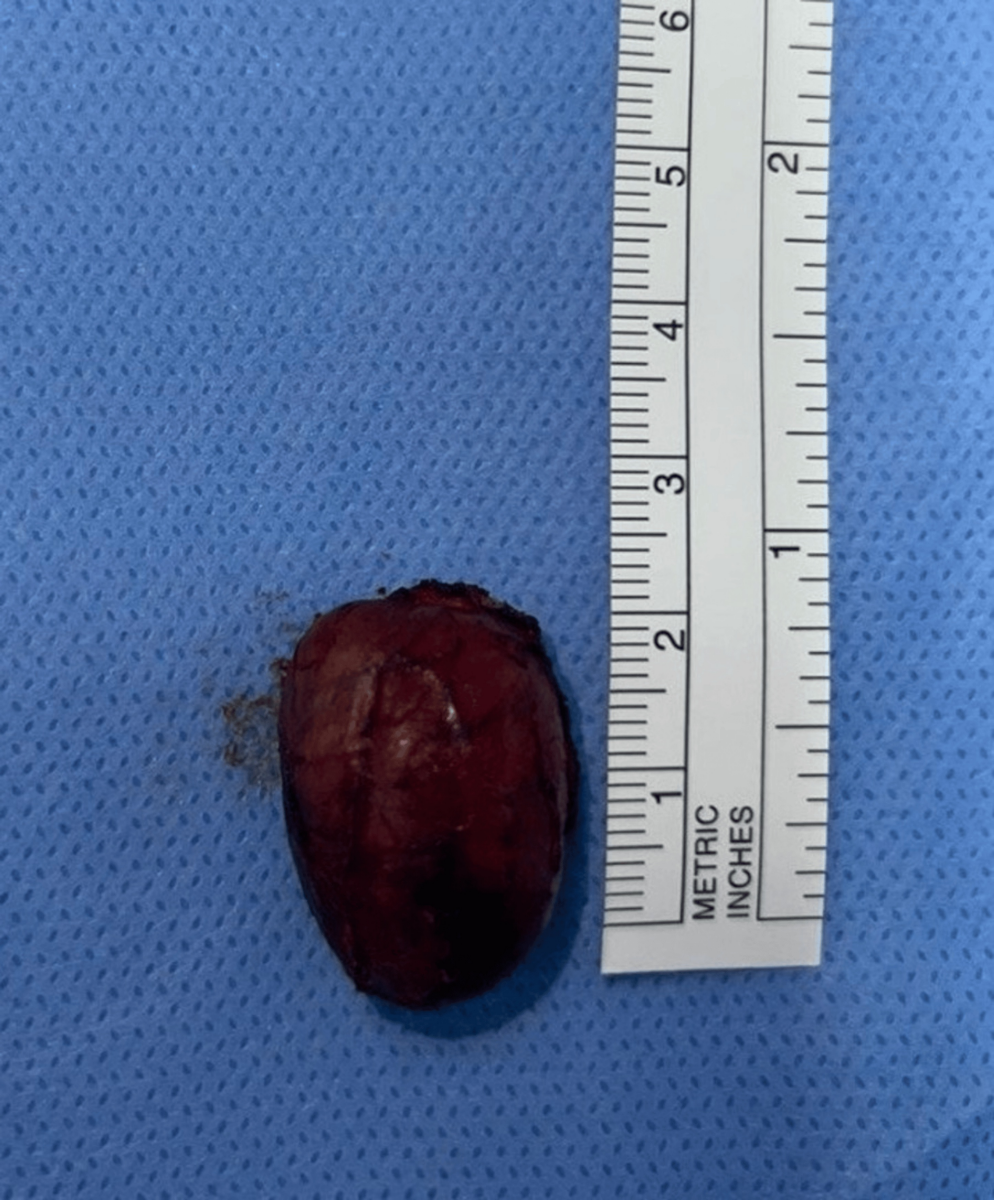
Excised cystic mass measuring approximately 4x3 cm, with intact capsule and smooth external surface.

Radiological assessment with a non-contrast computed tomography (CT) scan of the temporal bone demonstrated a well-defined subcutaneous cystic lesion located in the right postauricular region, with no evidence of bony erosion or intracranial extension (Figures 2a, 2b). Based on these findings, the patient underwent complete surgical excision of the mass under general anesthesia (Figures 3, 4).

**Figure 5:**
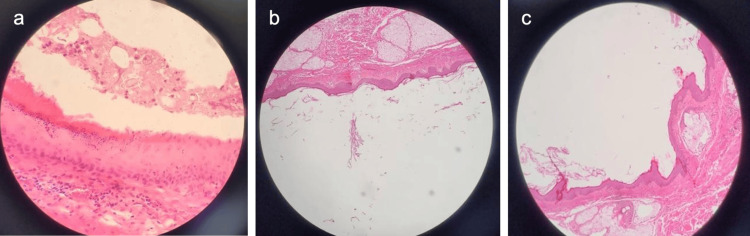
Histopathological features of the excised lesion (H&E stain). The images show (a) keratinized stratified squamous epithelial lining and adjacent sebaceous glands, (b) dermis containing multiple sebaceous glands, and (c) a hair follicle within the connective tissue wall, all consistent with a dermoid cyst.

The excised specimen was subsequently submitted to the histopathology laboratory for evaluation. On gross examination, it measured approximately 4×3×2 cm and revealed a cystic lesion containing sebaceous material and hair. Microscopically, the cyst wall was lined by keratinized stratified squamous epithelium with adnexal structures such as sebaceous glands and hair follicles, with no cellular atypia or malignant features observed (Figures 5a-5c). Histopathological analysis confirmed the diagnosis of a dermoid cyst. At the 36-month follow-up, the patient reported no signs of recurrence or other postoperative complications. On physical examination, the surgical site exhibited complete wound healing with a satisfactory cosmetic outcome.

## Discussion

Dermoid cysts of the auricle or periauricular region should be differentiated from other benign lesions such as epidermoid cysts, cystic teratomas, lipomas, branchial cysts, and trichilemmal cysts [4]. Table 1 summarizes the key distinguishing features of these entities, based on data from relevant case reports and other sources [1,5-7].

**Table 1:** Comparison of differential diagnoses of postauricular masses. SCC: squamous cell carcinoma; BCC: basal cell carcinoma

Lesion	Origin	Usual location	Presentation	Histopathology
Epidermoid cyst	Ectoderm	Scalp, neck, and postauricular (1-7%)	Dome-shaped, firm or fluctuant, may inflame or rupture	Stratified squamous epithelium with keratin, no adnexal structures
Dermoid cyst	Ectoderm and mesoderm	Midline; rarely postauricular	Slow-growing, well-defined	Stratified squamous epithelium with adnexal structures
Pilar (trichilemmal) cyst	A keratin-filled cyst that originates from the outer hair root sheath	Scalp, postauricular hair-bearing areas	Firm, thick, often multiple. Common in women	Abrupt keratinization without a granular layer
Branchial cleft cyst	First branchial cleft	Preauricular is more common than postauricular	Smooth, painless lateral cyst	Lined by squamous epithelium and lymphoid tissue
Lipoma	Mesenchymal (adipose)	15% occur in the head and neck region	Soft, mobile, painless. Slips under palpation	Lobules of mature adipocytes within a fibrous capsule
Lymphadenopathy (inflammatory)	Lymphoid	Postauricular lymph nodes	Tender, mobile, associated with infection	Reactive follicular hyperplasia
Cystic teratoma	All three germ layers	Midline; rarely postauricular	Congenital, solid cystic mass	Contains derivatives of the three germ layers (teeth, cartilage, and muscle)
Malignant neoplasms (SCC, BCC, lymphoma)	Epithelial or lymphoid	Postauricular, scalp, or parotid	Hard, irregular, fixed mass. Associated with facial palsy or constitutional symptoms	SCC: atypical keratinocytes. Lymphoma: atypical lymphoid cell

Dermoid cysts are classified as congenital or acquired [1]. The exact mechanism of their origin remains unknown; however, several theories have been proposed, including congenital incorporation of epithelial and dermal components of germ layer along the anatomical lines of embryonic closure, post-traumatic displacement of epithelial tissue, and development from displaced totipotent stem cells [1].

Dermoid cysts can develop in various anatomical locations. They most frequently arise in the gonads but may also appear at extragonadal midline sites. In the head and neck region, they are uncommon in both children and adults [5] and typically arise along embryonic lines of fusion, such as the midline of the neck, nasal region, nasolabial fold, or the lateral third of the eyebrow. Within the temporal bone, dermoid cysts have been reported in several locations, including the middle ear, mastoid process, Eustachian tube, and petrous apex [6].

A variety of diagnostic modalities are used in the evaluation of dermoid cysts. Ultrasound (US) is commonly employed as the initial modality because it is rapid, cost-effective, and radiation-free. Dermoid cysts typically appear as well-demarcated, homogeneous mass, hypoechoic cystic lesions. However, sonography alone cannot determine the lesion's extent or confirm the diagnosis [6]. Computed tomography (CT) is valuable for diagnostic confirmation and assessment of bony involvement, typically showing a well-defined, lobulated, fat-density mass that may contain calcifications [5]. Magnetic resonance imaging (MRI) offers enhanced soft-tissue characterization and is preferred for evaluating potential intracranial extension. On MRI, dermoid cysts demonstrate T1 hyperintensity from lipid content, variable T2 signal, and lack of enhancement after gadolinium administration. In contrast to epidermoid cysts, they rarely show restricted diffusion on diffusion-weighted imaging (DWI) [6].

Fine-needle aspiration cytology (FNAC) can also aid in differentiating dermoid cysts from other lesions, such as sebaceous cysts, lymphadenopathies, lipomas, and hemangiomas [2]. However, the definitive diagnosis relies on histopathological examination (HPE), which typically reveals a cyst lumen filled with keratin and hair shafts, lined by stratified squamous epithelium with mature skin adnexa such as hair follicles and sebaceous glands [2].

Complete surgical excision is the standard management for dermoid cysts, regardless of their location. Although many lesions are asymptomatic, excision is often performed for cosmetic purposes or to prevent future complications. When excision is undertaken, meticulous removal of the entire cyst wall and its contents is crucial, as incomplete removal may lead to infection or recurrence [2].

The overall prognosis of dermoid cysts in the head and neck region is excellent, particularly when the cyst is excised completely without rupture or residual tissue, and when there is no intracranial or intraspinal involvement or malignant transformation [2]. Recurrence has been reported in up to 20% of cases, particularly when excision is performed after cyst infection [2].

Table 2 summarizes postauricular dermoid cyst cases reported in the literature over the last decade, with only 10 cases identified, most involving young adults and showing a slight female predominance. The lesions were typically presented as unilateral, painless, long-standing swelling in the postauricular region. All patients underwent complete surgical excision, and follow-up results consistently demonstrated excellent cosmetic outcomes with no evidence of recurrence. These findings in the literature highlight the benign nature of postauricular dermoid cysts and the effectiveness of total removal as the definitive management.

**Table 2:** Summary of postauricular dermoid cyst cases (2015-2025).

S. no.	Year	Studies	Country	Patient age and gender	Presenting symptoms	Duration of symptoms	Treatment	Outcomes
1	2025	Khan et al. [2]	India	17 M	Left postauricular swelling	Since childhood	Surgical excision	No recurrence; follow-up duration not reported
2	2024	Mendonca et al. [7] (Case 1)	India	32 F	Left postauricular swelling	2 years	Surgical excision	Outcome not reported
3	2022	Aljeaid et al. [1]	Saudi Arabia	28 F	Right postauricular swelling	2 years	Surgical excision	No recurrence during 15-month follow-up
4	2022	Kharche and Iyer [3]	India	15 F	Left postauricular swelling	Since childhood	Surgical excision	No recurrence during 6-month follow-up
5	2021	Alberici et al. [6]	Italy	22 F	Right postauricular swelling	Since childhood	Surgical excision	No recurrence during 22-month follow-up
6	2020	Jeong et al. [4]	South Korea	31 F	Left postauricular swelling	10 years	Surgical excision	No recurrence during 9-month follow-up
7	2020	Kathuria et al. [8]	India	30 F	Left postauricular swelling	6 months	Surgical excision	No recurrence during 6-month follow-up
8	2018	Byeon et al. [9]	South Korea	19 M	Right postauricular swelling	Since childhood	Surgical excision	No recurrence during 6-month follow-up
9	2018	Duran [10]	Turkey	21 M	Right postauricular swelling	Since childhood	Surgical excision and otoplasty	Outcome not reported
10	2017	Wisecarver et al. [11]	USA	2 M	Right postauricular swelling	Since childhood	Surgical excision	Outcome not reported

## Conclusions

Postauricular dermoid cysts are extremely rare and may remain asymptomatic for long periods, although they typically grow slowly over time. Surgical excision is generally recommended, particularly for cosmetic reasons, as removal at an early stage can improve cosmetic outcomes and reduce the risk of infection. Following surgical excision, histopathological examination is mandatory to confirm the diagnosis and exclude the possibility of rare malignant transformation. This case demonstrates the significance of including dermoid cysts in the differential diagnosis of postauricular swellings, as they can mimic other benign head and neck lesions.
